# Supplementary material for: Two complement receptor one alleles have opposing associations with cerebral malaria and interact with α+thalassaemia
Source: eLife. 2018 Apr 25;7:e31579. doi: 10.7554/eLife.31579 (PMC5953541; doi:10.7554/eLife.31579)
Supplement: Supplementary file 1. — (A) General characteristics for the Kenyan case-control study. (B) Characteristics for the Kenyan case-control study by Sl and McC genotype. (C) Hardy Weinberg equilibrium calculations for controls in the Kenyan case-control study. (D) Unadjusted odds ratios for clinical outcomes for the Kenyan case-control study. (E) Sl and McC combined genotypes and adjusted odds ratios for cerebral malaria in the Kenyan case-control study. (F) Sl and McC combined genotypes and adjusted odds ratio for death in the Kenyan case-control study. (G) General characteristics of the Kenyan longitudinal cohort study population by Sl and McC genotypes. (H) Incidence of common childhood diseases by Sl genotypes in the Kenyan longitudinal cohort study. (I) Incidence of common childhood diseases by McC genotypes in the Kenyan longitudinal cohort study. (J) Unadjusted Incidence Rate Ratios (IRR) for uncomplicated malaria and non-malarial diseases in the Kenyan longitudinal cohort study by Sl and McC genotype. (K) Reanalysis of Kenyan case-control study including children who lived outside of the KHDSS study area. (L) Adjusted Odds Ratios for different genetic models for the Sl polymorphism in the Kenyan case-control study. (M) Adjusted Odds Ratios for different genetic models for the McC polymorphism in the Kenyan case-control study. (N) Investigation of the sickle trait/α+thalassaemia negative epistatic interaction and the Sl2/α+thalassaemia interaction by clinical outcome in the Kenyan case-control study. (O) Reanalysis of the Kenyan case-control study excluding all children with one or more sickle cell alleles. (P) Raw data for the combined sickle trait, α+thalassaemia and Sl genotype by clinical outcome in the Kenyan case-control study. (Q) Correlations between the sickle cell, α + thalassaemia, Sl2 and McCb variants in the Kenyan case-control study. (R) Adjusted incidence Rate Ratios (IRRs) for Sl disease associations in the longitudinal cohort study by genetic models of inheritance (S) Ad [file elife-31579-supp1.docx]

## **Supplementary File 1**

## **A. General characteristics for the Kenyan case-control study.**

|  | Controls | Cases | p value |
| --- | --- | --- | --- |
| Kenya | n = 3829 | n = 1716 (all severe malaria) |  |
| Gender |  |  |  |
| Males | 1931 (50.4 %) | 862 (50.2 %) |  |
| Females | 1898 (49.6 %) | 854 (49.8 %) | 0.915 |
|  |  |  |  |
| Ethnicity |  |  |  |
| Giriama | 1775 (46.4 %) | 953 (55.5 %) |  |
| Chonyi | 1365 (35.6 %) | 435 (25.4 %) |  |
| Kauma | 440 (11.5 %) | 148 (8.6 %) |  |
| Others | 249 (6.5 %) | 180 (10.5 %) | **<0.001** |
|  |  |  |  |
| Age in months*  Median (IQR) | 6 (5-8) | 28 (15-43) | **<0.001** |

Comparisons performed using Pearson’s χ^2^ test except *Kruskal Wallis test.

**B. Characteristics for the Kenyan case-control study by *Sl* and *McC* genotype.**

|  |  | ***Sl1/Sl1*** | ***%*** | ***Sl1/Sl2*** | ***%*** | | ***Sl2/Sl2*** | ***%*** | **p value** | |  | ***McC^a^/ McC^a^*** | ***%*** | ***McC^a^/McC^b^*** | ***%*** | ***McC^b^/ McC^b^*** | ***%*** | **p value** |
| --- | --- | --- | --- | --- | --- | --- | --- | --- | --- | --- | --- | --- | --- | --- | --- | --- | --- | --- |
| **Clinical status** | Control | 378 | *9.9* | 1730 | *45.2* | | 1721 | *44.9* |  | |  | 2700 | *70.5* | 1040 | *27.2* | 89 | *2.3* |  |
|  | All severe cases | 177 | *10.3* | 801 | *46.7* | | 738 | *43.0* | 0.403 | |  | 1182 | *68.9* | 484 | *28.2* | 50 | *2.9* | 0.278 |
|  | CM | 95 | *10.1* | 457 | *48.5* | | 391 | *41.5* | | 0.144 |  | 633 | *67.1* | 284 | *30.1* | 26 | *2.8* | 0.122 |
|  | Severe no CM | 71 | *10.5* | 297 | *44.1* | | 306 | *45.4* | | 0.804 |  | 479 | *71.1* | 175 | *26.0* | 20 | *3.0* | 0.520 |
|  | SMA | 53 | *11.0* | 230 | *47.6* | 200 | | *41.4* | | 0.319 |  | 346 | *71.6* | 128 | *26.5* | 9 | *1.9* | 0.761 |
|  | SMA no CM | 27 | *12.1* | 100 | *44.8* | | 96 | *43.0* | | 0.541 |  | 162 | *72.6* | 56 | *25.1* | 5 | *2.2* | 0.791 |
|  | RD | 54 | *10.3* | 245 | *46.9* | | 223 | *42.7* | | 0.630 |  | 355 | *68.0* | 153 | *29.3* | 14 | *2.7* | 0.488 |
|  | RD no CM | 24 | *12.5* | 80 | *41.7* | | 88 | *45.8* | | 0.407 |  | 132 | *68.8* | 54 | *28.1* | 6 | *3.1* | 0.626^#^ |
|  | Died | 25 | *13.9* | 80 | *44.4* | | 75 | *41.7* | | 0.203 |  | 119 | *66.1* | 53 | *29.4* | 8 | *4.4* | 0.128^#^ |
|  | Died with CM | 16 | *12.2* | 59 | *45.0* | | 56 | *42.7* | | 0.659 |  | 83 | *63.4* | 44 | *33.6* | 4 | *3.1* | 0.184^#^ |
|  | Died no CM | 8 | *19.0* | 18 | *42.9* | | 16 | *38.1* | | 0.148^#^ |  | 31 | *73.8* | 9 | *21.4* | 2 | *4.8* | 0.304^#^ |
|  | Died with SMA | 9 | *16.1* | 24 | *42.9* | | 23 | *41.1* | | 0.304 |  | 38 | *67.9* | 14 | *25.0* | 4 | *7.1* | 0.088^#^ |
|  | Died with RD | 10 | *13.7* | 34 | *46.6* | | 29 | *39.7* | | 0.467 |  | 51 | *69.9* | 21 | *28.8* | 1 | *1.4* | 0.934^#^ |
| **Gender** | M | 270 | *9.7* | 1287 | *46.1* | | 1236 | *44.3* | |  |  | 1928 | *69.0* | 802 | *28.7* | 63 | *2.3* |  |
|  | F | 285 | *10.4* | 1244 | *45.2* | | 1223 | *44.4* | | 0.637 |  | 1954 | *71.0* | 722 | *26.2* | 76 | *2.8* | 0.071 |
| **Ethnicity** | Giriama | 235 | *8.6* | 1223 | *44.8* | | 1270 | *46.6* | |  |  | 1881 | *69.0* | 777 | *28.5* | 70 | *2.6* |  |
|  | Chonyi | 204 | *11.3* | 832 | *46.2* | | 764 | *42.4* | |  |  | 1307 | *72.6* | 462 | *25.7* | 31 | *1.7* |  |
|  | Kauma | 68 | *11.6* | 279 | *47.4* | | 241 | *41.0* | |  |  | 410 | *69.7* | 160 | *27.2* | 18 | *3.1* |  |
|  | Other | 48 | *11.2* | 197 | *45.9* | | 184 | *42.9* | | **0.007** |  | 284 | *66.2* | 125 | *29.1* | 20 | *4.7* | **0.003** |
| **Sickle** | Hb AA | 489 | *10.0* | 2234 | *45.8* | | 2158 | *44.2* | |  |  | 3404 | *69.7* | 1348 | *27.6* | 129 | *2.6* |  |
|  | Hb AS | 60 | *9.6* | 282 | *45.3* | | 280 | *45.0* | |  |  | 444 | *71.4* | 168 | *27.0* | 10 | *1.6* |  |
|  | Hb SS | 6 | *14.3* | 15 | *35.7* | | 21 | *50.0* | | 0.66^#^ |  | 34 | *81.0* | 8 | *19.0* | 0 | *0.0* | 0.311^#^ |
| **α+thalassaemia** | α α | 189 | *9.5* | 912 | *45.8* | | 890 | *44.7* | |  |  | 1398 | *70.2* | 551 | *27.7* | 42 | *2.1* |  |
|  | α -α | 279 | *10.3* | 1244 | *45.8* | | 1196 | *44.0* | |  |  | 1898 | *69.8* | 739 | *27.2* | 82 | *3.0* |  |
|  | -α -α | 87 | *10.4* | 375 | *44.9* | | 373 | *44.7* | | 0.892 |  | 586 | *70.2* | 234 | *28.0* | 15 | *1.8* | 0.200 |
| **Blood group** | O | 287 | *9.8* | 1328 | *45.5* | | 1302 | *44.6* | |  |  | 2042 | *70.0* | 799 | *27.4* | 76 | *2.6* |  |
|  | Non-O | 268 | *10.2* | 1203 | *45.8* | | 1157 | *44.0* | | 0.855 |  | 1840 | *70.0* | 725 | *27.6* | 63 | *2.4* | 0.879 |
| **Median age** | Months | 8 | IQR  5-18 | 8 | IQR  5-13 | | 8 | IQR  5-12 | | 0.636 |  | 8 | IQR  5-12 | 8 | IQR  5-14 | 8 | IQR  5-18.5 | 0.441 |

CM, cerebral malaria; SMA, severe malarial anaemia; RD, respiratory distress; IQR, interquartile range. Significance testing for each clinical status uses control as the reference group. Age: Kruskal Wallis test. ^#^: Fisher’s exact test. All other analyses: Pearson’s χ^2^.

**C. Hardy Weinberg equilibrium calculations for controls in the Kenyan case-control study.**

| ***Sl1/Sl1*** | ***Sl1/Sl2*** | ***Sl2/Sl2*** | **χ2** | **p value** |
| --- | --- | --- | --- | --- |
| 378 | 1730 | 1721 | 3.43 | 0.064* |
|  |  |  |  |  |
| ***McC^a^/McC^a^*** | ***McC^a^/McC^b^*** | ***McC^b^/McC^b^*** | **χ2** | **p value** |
| 2700 | 1040 | 89 | 0.81 | 0.369 |

*Although *Sl* and *McC* were found to be in Hardy Weinberg Equilibrium (HWE), *Sl* was of borderline statistical significance. HWE calculations for *Sl* were stratified by ethnicity and found to deviate from HWE for the Giriama ethnicity (χ2 = 5.12, p = 0.024), but not for the other ethnic groups. Ethnicity was subsequently included as a variable in all analyses performed.

To further investigate whether a genotyping error could be responsible for the borderline significant deviation from HWE for *Sl* in the Kenyan population, a subset of samples (n= 2344) were genotyped using a second platform (Illumina HumanOmni2.5-4) as previously described [1]. The concordancy rates between the two methods were 98.3 % (2304/2344 samples) for *Sl* and 98.8 % (2317/2344) for *McC*.

**Reference**

1.         Malaria Genomic Epidemiology Network. A novel locus of resistance to severe malaria in a region of ancient balancing selection. Nature. 2015;526: 253–257.

**D. Unadjusted odds ratios for clinical outcomes for the Kenyan case-control study.**

| **Clinical Outcome** | ***Sl2/Sl2***  **Unadjusted OR (95% CI)** | **p value** | ***McC^b^***  **Unadjusted OR (95% CI)** | **p value** |
| --- | --- | --- | --- | --- |
| **Controls**  **(n= 3829)** | Ref |  | Ref |  |
| **All severe malaria (n=1716)** | 0.90 (0.79 – 1.01) | 0.071 | 1.17 (1.00 – 1.25) | 0.056 |
| **CM (n=943)** | **0.82 (0.71 – 0.95)** | **0.010** | **1.21 (1.05 – 1.39)** | **0.008** |
| **Severe without CM (n=674)** | 1.09 (0.86 – 1.21) | 0.829 | 1.00 (0.84 – 1.18) | 0.985 |
| **Died (n=180)** | 0.80 (0.58 – 1.09) | 0.164 | **1.34 (1.00 – 1.77)** | **0.046** |
| **Died with CM (n=131)** | 0.82 (0.57 – 1.19) | 0.306 | 1.39 (0.99 - 1.92) | 0.052 |
| **Died without CM (n=42)** | 0.74 (0.38 – 1.41) | 0.373 | 1.05 (0.54 – 1.91) | 0.881 |
| **SMA(n=483)** | 0.87 (0.71 – 1.06) | 0.177 | 0.98 (0.80 – 1.18) | 0.800 |
| **SMA without CM (n=223)** | 0.94 (0.71 – 1.25) | 0.693 | 0.93 (0.70 – 1.23) | 0.620 |
| **Died with SMA (n=56)** | 0.77 (0.43 – 1.24) | 0.327 | 1.40 (0.83 – 2.27) | 0.192 |
| **RD^§^ (n=522)** | 0.88 (0.72 – 1.06) | 0.181 | 1.16 (0.96 – 1.38) | 0.129 |
| **RD without CM (n=192)** | 1.01 (0.74 – 1.37) | 0.955 | 1.10 (0.82 – 1.45) | 0.529 |
| **Died with RD (n=73)** | 0.80 (0.48 – 1.29) | 0.363 | 1.05 (0.64 – 1.66) | 0.834 |

Model includes only *Sl2/Sl2* in recessive form (i.e. vs *Sl1/Sl1* and *Sl1/Sl2*) and *McC^b^* in additive form (i.e. impact of each additional *McC^b^* allele). The data are not adjusted for the potential confounders ethnic group, location, sickle cell trait, ABO blood group and α^+^thalassaemia genotype. Confidence intervals by Wald. The 95% CIs and p values are not bootstrapped.

OR = odds ratio; CI = confidence interval; CM = cerebral malaria; SMA = severe malarial anaemia; RD, respiratory distress. 34/56 cases who died with SMA also had CM. 56/73 cases who died with RD also had CM.

**E. *Sl* and *McC* combined genotypes and adjusted odds ratios for cerebral malaria in the Kenyan case-control study.**

| Genotype | Number of controls/ CM cases | aOR | 95% CI | p value |
| --- | --- | --- | --- | --- |
| *Sl1/Sl- McC^a^/McC^a^* | 378/95 | Ref | Ref | Ref |
| *Sl1/Sl2- McC^a^/McC^a^* | 1330/322 | 0.89 | 0.59 - 1.41 | 0.622 |
| *Sl1/Sl2- McC^a^/McC^b^* | 400/135 | 1.15 | 0.69 - 1.92 | 0.606 |
| *Sl2/Sl2- McC^a^/McC^a^* | 992/216 | 0.70 | 0.44 – 1.11 | 0.123 |
| ***Sl2/Sl2-McC^a^/McC^b^*** | **640/149** | **0.57** | **0.33 – 0.96** | **0.031** |
| *Sl2/Sl2- McC^b^/McC^b^* | 89/26 | 1.12 | 0.38 – 2.76 | 0.749 |

aOR: adjusted Odds Ratio. aORs were adjusted for ethnicity, location of residence, sickle cell genotype, α+thalassaemia genotype and ABO blood group. An interaction term between the combined *Sl/McC* genotype and α+thalassaemia was included in the model. Model outputs following 2000 bootstrapped iterations are displayed.

**F. *Sl* and *McC* combined genotypes and adjusted odds ratio for death in the Kenyan case-control study.**

|  | Number survived/died | aOR | 95% CI | p value |
| --- | --- | --- | --- | --- |
| *Sl1/Sl1-McC^a^/McC^a^* | 378/25 | Ref | Ref | Ref |
| *Sl1/Sl2-McC^a^/McC^a^* | 1330/57 | 0.73 | 0.35 – 1.77 | 0.383 |
| *Sl1/Sl2-McC^a^/McC^b^* | 400/23 | 1.04 | 0.43 – 2.72 | 0.946 |
| *Sl2/Sl2-McC^a^/McC^a^* | 992/37 | 0.44 | 0.19 – 1.10 | 0.080 |
| ***Sl2/Sl2-McC^a^/McC^b^*** | **640/30** | **0.34** | **0.13 – 0.92** | **0.034** |
| *Sl2/Sl2-McC^b^/McC^b^* | 89/8 | 1.17 | 0.00 – 6.09 | 0.530 |

aOR: adjusted Odds Ratio. aORs were adjusted for ethnicity, location of residence, sickle cell genotype, α+thalassaemia genotype and ABO blood group. An interaction term between the combined *Sl/McC* genotype and α+thalassaemia is included in the model. Model outputs following 2000 bootstrapped iterations are displayed.

**G. General characteristics of the Kenyan longitudinal cohort study population by *Sl* and *McC* genotypes.**

| Characteristics |  | *Sl1/Sl1* | *%* | *Sl1/Sl2* | *%* | *Sl2Sl2* | *%* | *p value* | *McC^a^/*  *McC^a^* | *%* | *McC^a^/*  *McC^b^* | *%* | *McC^b^/*  *McC^b^* | *%* | *p value* |
| --- | --- | --- | --- | --- | --- | --- | --- | --- | --- | --- | --- | --- | --- | --- | --- |
|  |  |  |  |  |  |  |  |  |  |  |  |  |  |  |  |
| Sample size |  | 22 | *10.6* | 94 | *45.2* | 92 | *44.2* |  | 137 | *65.9* | 63 | *30.2* | 8 | *3.9* |  |
| Age in months  Mean (95% CI) |  | 18(14-44) |  | 32(13-43) |  | 23(8-48) |  | 0.609 | 25(11-45) |  | 31(13-49) |  | 12(5-21) |  | 0.112 |
| Gender |  |  |  |  |  |  |  |  |  |  |  |  |  |  |  |
|  | Males | 13 | *10.8* | 56 | *46.7* | 51 | *42.5* |  | 82 | *68.3* | 33 | *27.5* | 5 | *4.2* |  |
|  | Females | 9 | *10.2* | 38 | *43.2* | 41 | *46.6* | 0.844 | 55 | *62.5* | 30 | *34.1* | 3 | *3.4* | 0.594 |
| Ethnic group |  |  |  |  |  |  |  |  |  |  |  |  |  |  |  |
|  | Giriama | 19 | *10.8* | 80 | *45.5* | 77 | *43.8* |  | 113 | *64.2* | 55 | *31.3* | 8 | *4.6* |  |
|  | Chonyi | 2 | *10.0* | 10 | *50.0* | 8 | *40.0* |  | 15 | *75.0* | 5 | *25.0* | 0 | *0.0* |  |
|  | Others | 1 | *8.3* | 4 | *33.3* | 7 | *58.3* | 0.907 | 9 | *75.0* | 3 | *25.0* | 0 | *0.0* | 0.877 |
| Sickle |  |  |  |  |  |  |  |  |  |  |  |  |  |  |  |
|  | AA | 17 | *9.3* | 85 | *46.7* | 80 | *44.0* |  | 122 | *67.0* | 52 | *28.6* | 8 | *4.4* |  |
|  | AS | 5 | *19.2* | 9 | *34.6* | 12 | *46.2* | 0.224 | 15 | *57.7* | 11 | *42.3* | 0 | *0.0* | 0.228 |
| α+thalassaemia |  |  |  |  |  |  |  |  |  |  |  |  |  |  |  |
|  | Normal (αα/αα) | 7 | *10.5* | 33 | *49.3* | 27 | *40.3* |  | 45 | *67.2* | 18 | *26.9* | 4 | *6.0* |  |
|  | Heterozygote -α/αα | 9 | *8.7* | 48 | *46.2* | 47 | *45.2* |  | 67 | *64.4* | 34 | *32.7* | 3 | *2.9* |  |
|  | Homozygote -α/-α | 6 | *16.2* | 13 | *35.1* | 18 | *48.7* | 0.546 | 25 | *67.6* | 11 | *29.7* | 1 | *2.7* | 0.823 |
| ABO blood group |  |  |  |  |  |  |  |  |  |  |  |  |  |  |  |
|  | O | 11 | *9.4* | 56 | *47.9* | 50 | *42.7* |  | 81 | *69.2* | 30 | *25.6* | 6 | *5.1* |  |
|  | Non O | 11 | *12.1* | 38 | *41.8* | 42 | *46.1* | 0.628 | 56 | *61.5* | 33 | *36.3* | 2 | *2.2* | 0.189 |

All comparisons were done using the Fisher’s exact test.

**H. Incidence of common childhood diseases by *Sl* genotypes in the Kenyan longitudinal cohort study.**

|  | *Sl1/Sl1* | | | *Sl1/Sl2* | | | *Sl2/Sl2* | |
| --- | --- | --- | --- | --- | --- | --- | --- | --- |
| Clinical  Outcomes | No. of episodes | Incidence | No. of episodes | | Incidence | No. of episodes | | Incidence |
|  |  |  |  | |  |  | |  |
| All non-malaria clinic visits | 285 | 5.77 | 1052 | | 4.92 | 1020 | | 5.40 |
|  |  |  |  | |  |  | |  |
| Uncomplicated malaria | 124 | 2.51 | 493 | | 2.31 | 461 | | 2.44 |
|  |  |  |  | |  |  | |  |
| LRTI^†^ | 46 | 0.93 | 180 | | 0.84 | 172 | | 0.91 |
|  |  |  |  | |  |  | |  |
| URTI^¶^ | 111 | 2.25 | 394 | | 1.84 | 403 | | 2.13 |
|  |  |  |  | |  |  | |  |
| Gastroenteritis | 64 | 1.30 | 158 | | 0.74 | 145 | | 0.77 |
|  |  |  |  | |  |  | |  |
| Skin infection | 37 | 0.75 | 196 | | 0.92 | 176 | | 0.93 |
|  |  |  |  | |  |  | |  |
| Helminth infection | 7 | 0.14 | 52 | | 0.24 | 44 | | 0.23 |
|  |  |  |  | |  |  | |  |
| Malaria negative fever | 104 | 2.11 | 301 | | 1.41 | 233 | | 1.23 |

Incidence = number of episodes per child-year of follow up. Data were collected from 22 *Sl1/Sl1*, 94 *Sl1/Sl2* and 92 *Sl2/Sl2* individuals during 49.4, 213.8 and 188.8 cyfu (child-years of follow-up) respectively.

^†^LRTI: Lower Respiratory Tract Infection; ^¶^URTI: Upper Respiratory Tract Infection

**I. Incidence of common childhood diseases by *McC* genotypes in the Kenyan longitudinal cohort study.**

|  | *McC^a^/McC^a^* | | | *McC^a^/McC^b^* | | | *McC^b^/McC^b^* | |
| --- | --- | --- | --- | --- | --- | --- | --- | --- |
| Clinical  Outcomes | No. of episodes | Incidence | No. of episodes | | Incidence | No. of episodes | | Incidence |
|  |  |  |  | |  |  | |  |
| All non-malaria clinic visits | 1595 | 5.42 | 685 | | 4.78 | 77 | | 5.38 |
|  |  |  |  | |  |  | |  |
| Uncomplicated malaria | 699 | 2.37 | 354 | | 2.47 | 25 | | 1.75 |
|  |  |  |  | |  |  | |  |
| LRTI^†^ | 273 | 0.93 | 117 | | 0.82 | 8 | | 0.56 |
|  |  |  |  | |  |  | |  |
| URTI^¶^ | 624 | 2.12 | 246 | | 1.72 | 38 | | 2.66 |
|  |  |  |  | |  |  | |  |
| Gastroenteritis | 252 | 0.86 | 101 | | 0.71 | 14 | | 0.98 |
|  |  |  |  | |  |  | |  |
| Skin infection | 261 | 0.89 | 141 | | 0.98 | 7 | | 0.49 |
|  |  |  |  | |  |  | |  |
| Helminth infection | 74 | 0.25 | 28 | | 0.20 | 1 | | 0.07 |
|  |  |  |  | |  |  | |  |
| Malaria negative fever | 423 | 1.43 | 192 | | 1.34 | 23 | | 1.61 |

Incidence = number of episodes per child-year of follow up. Data were collected from 137 *McC^a^/McC^a^*, 63 *McC^a^*/*McC^b^* and 8 *McC^b^/McC^b^* individuals during 294.5, 143.2 and 14.3 cyfu respectively.

^†^LRTI: Lower Respiratory Tract Infection; ^¶^URTI: Upper Respiratory Tract Infection

**J. Unadjusted Incidence Rate Ratios (IRR) for uncomplicated malaria and non-malarial diseases in the Kenyan longitudinal cohort study by *Sl* and *McC* genotype.**

| Clinical  Outcomes | *Sl2*  IRRs^§^ (95% CI) | *p* value | *McC^b^*  IRRs (95% CI) | *p* value |
| --- | --- | --- | --- | --- |
|  |  |  |  |  |
| Uncomplicated malaria | 0.95 (0.76-1.18) | 0.638 ^4^ | 0.72 (0.32-1.63) | 0.254 **^1^** |
|  |  |  |  |  |
| All non-malaria clinical visits | 1.03 (0.87-1.22) | 0.711 **^1^** | 0.91 (0.79-1.06) | 0.242 **^4^** |
|  |  |  |  |  |
| LRTI^†^ | 1.02 (0.74-1.41) | 0.902 **^1^** | 0.54 (0.21-1.43) | 0.217 ^1^ |
|  |  |  |  |  |
| URTI^¶^ | 1.08 (0.88-1.33) | 0.472 **^1^** | 0.81 (0.65-1.02) | 0.077 **^3^** |
|  |  |  |  |  |
| Gastroenteritis | **0.56 (0.34-0.95)** | **0.031 ^2^** | 0.87 (0.60-1.26) | 0.462 **^2^** |
|  |  |  |  |  |
| Skin infection | 1.24 (0.73-2.12) | 0.430 **^2^** | 0.47 (0.17-1.29) | 0.142 **^1^** |
|  |  |  |  |  |
| Helminth infection | 1.63 (0.69-3.86) | 0.265 **^2^** | 0.72 (0.46-1.12) | 0.147 **^4^** |
|  |  |  |  |  |
| Malaria negative fever | 0.70 (0.49-1.02) | 0.060 **^2^** | 0.91 (0.70-1.19) | 0.486 **^3^** |

Data were collected from 22 *Sl1/Sl1*, 94 *Sl1/Sl2* and 92 *Sl2/Sl2* individuals during 49.4, 213.8 and 188.8 cyfu (child-years of follow-up) respectively and 137 *McC^a^/McC^a^*, 63 *McC^a^*/*McC^b^* and 8 *McC^b^/McC^b^* individuals during 294.5, 143.2 and 14.3 cyfu respectively. *Sl2* and *McC^b^* alleles were tested separately in univariate analyses for their association with the disease outcomes of interest using logistic regression in the **^1^**recessive, **^2^**dominant, **^3^**heterozygous and **^4^**additive models. The best fitting models as examined using the Akaike information criterion (AIC) were used in the final analysis that accounted for within-person clustering of events. ^§^IRRs: (unadjusted) Incidence Rate Ratios**;** ^†^LRTI: Lower Respiratory Tract Infection**;** ^¶^URTI: Upper Respiratory Tract Infection;

**K. Reanalysis of Kenyan case-control study including children who lived outside of the KHDSS study area.**

| **Clinical Outcome** | ***Sl2***  **aOR (95% CI)** | ***p value*** | ***McCb***  **aOR (95% CI)** | ***p value*** |
| --- | --- | --- | --- | --- |
| **All severe malaria**  **(n= 2100)** | 0.84  (0.70 - 1.01) | 0.069 | 1.07  (0.96 - 1.19) | 0.232 |
| **CM**  **(n=1162)** | **0.72**  **(0.57 – 0.90)** | **0.004** | **1.18**  **(1.03 – 1.34)** | **0.016** |
| **Severe without CM**  **(n=805)** | 1.05  (0.82 – 1.35) | 0.672 | 0.967  (0.83 – 1.13) | 0.678 |
| **Died**  **(n= 241** | 0.69  (0.45 – 1.05) | 0.083 | 1.16  (0.90 – 1.49) | 0.261 |
| **SMA**  **(n= 629)** | 0.95  (0.72 – 1.24) | 0.693 | 0.92  (0.77 – 1.09) | 0.335 |
| **RD**  **(n= 653)** | 0.87  (0.66 – 1.15) | 0.333 | 1.16  (0.98 – 1.36) | 0.086 |

Analysis of the dataset including the 386 children who lived outside of the KHDSS study area (407 children lived outside the KHDSS but only 386 had full data on sickle cell, α+thalassaemia, *Sl*, *McC* and ABO genotype, thus available for inclusion in this analysis.) These children were all severe malaria cases and were previously excluded from the main analysis as they did not have location-specific controls.

Adjusted Odds Ratios (aOR) and 95% Confidence Intervals (CI) are presented for the *Sl2* genotype in the recessive form (i.e. *Sl2/Sl2* vs all other *Sl* genotypes) and *McC^b^* genotype in the additive form (i.e. change in aOR with each additional *McC^b^* allele). aORs displayed are adjusted for ethnicity, sickle cell genotype, α+thalassaemia genotype and ABO blood group. An interaction term between *Sl* genotype and α+thalassaemia was included in the model. Confidence intervals by Wald. The 95% CIs and p values are not bootstrapped.

In contrast to the main results table in the manuscript, location of residence could not be used in this model as the newly included cases had no location-specific controls. Removal of the location variable resulted in deterioration of the model fits.

CM = cerebral malaria; SMA = severe malarial anaemia; RD = respiratory distress

**L. Adjusted Odds Ratios for different genetic models for the *Sl* polymorphism in the Kenyan case-control study.**

|  | **Sl2 recessive** | **Sl2 additive** | **Sl2 heterozygous** | **Sl2 dominant** | **Sl2 genotypic (Sl1/Sl1 = ref)**  **Sl1/Sl2 Sl2/Sl2** | |
| --- | --- | --- | --- | --- | --- | --- |
| **Case**  **(n=1716)**  aOR  95% CI  p value | **0.77**  **0.63-0.95**  **0.015** | **0.82**  **0.70-0.96**  **0.012** | 1.18  0.96-1.44  0.110 | 0.79  0.56-1.11  0.172 | 0.88  0.61-1.25  0.460 | **0.69**  **0.48-0.99**  **0.046** |
| **All CM**  **(n=943)**  aOR  95% CI  p value | **0.66**  **0.51-0.86**  **0.002** | **0.74**  **0.61-0.90**  **0.002** | **1.35**  **1.05-1.73**  **0.018** | 0.76  0.50-1.16  0.205 | 0.90  0.58-1.39  0.640 | **0.61**  **0.39-0.95**  **0.030** |
| **All SMA ^*^**  **(n=483)**  aOR  95% CI  p value | 0.77  0.56-1.05  0.101 | **0.77**  **0.60-0.97**  **0.027** | 1.06  0.78-1.45  0.702 | **0.61**  **0.38-0.98**  **0.040** | 0.66  0.40-1.08  0.099 | **0.54**  **0.33-0.91**  **0.020** |
| **All RD ^#^**  **(n= 522)**  aOR  95% CI  p value | 0.81  0.59-1.10  0.175 | 0.82  0.65-1.04  0.103 | 1.10  0.81-1.49  0.545 | 0.73  0.44-1.20  0.211 | 0.78  0.47-1.32  0.358 | 0.66  0.39-1.12  0.122 |
| **All Deaths (n=180)**  aOR  95% CI  p value | **0.50**  **0.30-0.83**  **0.007** | **0.59**  **0.41-0.84**  **0.004** | 1.53  0.96-2.45  0.076 | 0.54  0.27-1.10  0.090 | 0.72  0.35-1.50  0.378 | **0.38**  **0.17-0.82**  **0.015** |

The “base model” is kept the same throughout (ethnicity and location of residence as random effects; sickle cell genotype, α^+^thalassaemia genotype and ABO blood group as fixed effects. An interaction term for Sl * α^+^thalassaemia is included). The *McC^b^* allele was kept in the additive form for all analyses. CM = cerebral malaria; SMA = severe malarial anaemia; RD = respiratory distress.

^*^199 SMA cases also had CM. ^#^ 324 RD cases also had CM. CIs by Wald.

**M. Adjusted Odds Ratios for different genetic models for the *McC* polymorphism in the Kenyan case-control study.**

|  | **McC^b^ recessive** | **McC^b^ additive** | **McC^b^ heterozygous** | **McC^b^ dominant** | **McC^b^ genotypic (McC^a^ / McC^a^ = ref)**  **McC^a^ / McC^b^ McC^b^ / McC^b^** | |
| --- | --- | --- | --- | --- | --- | --- |
| **Case**  **(n=1716)**  aOR  95% CI  p value | 1.26  0.86-1.85  0.231 | 1.10  0.98-1.25  0.112 | 1.07  0.93-1.23  0.338 | 1.10  0.96-1.26  0.170 | 1.09  0.94-1.25  0.253 | 1.31  0.89-1.92  0.176 |
| **All CM**  **(n=943)**  aOR  95% CI  p value | 1.19  0.74-1.92  0.477 | **1.19**  **1.02-1.38**  **0.024** | **1.19**  **1.01-1.42**  **0.039** | **1.22**  **1.03-1.44**  **0.023** | **1.21**  **1.02-1.44**  **0.029** | 1.29  0.79-2.08  0.306 |
| **All SMA ^*^**  **(n=483)**  aOR  95% CI  p value | 0.83  0.40-1.70  0.611 | 0.96  0.78-1.18  0.690 | 0.99  0.79-1.24  0.913 | 0.97  0.77-1.21  0.782 | 0.98  0.78-1.23  0.859 | 0.82  0.40-1.70  0.598 |
| **All RD ^#^**  **(n= 522)**  aOR  95% CI  p value | 1.17  0.64-2.14  0.604 | 1.12  0.93-1.36  0.219 | 1.12  0.90-1.39  0.300 | 1.14  0.92-1.41  0.230 | 1.13  0.91-1.40  0.263 | 1.23  0.67-2.26  0.498 |
| **All Death**  **(n=180)**  aOR  95% CI  p value | 2.11  0.97-4.56  0.058 | 1.32  0.99-1.76  0.060 | 1.15  0.82-1.61  0.424 | 1.28  0.92-1.78  0.147 | 1.21  0.86-1.70  0.275 | **2.28**  **1.04-5.01**  **0.039** |

The “base model” is kept the same throughout (ethnicity and location of residence as random effects; sickle cell genotype, α^+^thalassaemia genotype and ABO blood group as fixed effects. An interaction term for Sl * α^+^thalassaemia is included). The *Sl2* allele was kept in the recessive form for all analyses. CM = cerebral malaria; SMA = severe malarial anaemia; RD = respiratory distress.

^*^199 SMA cases also had CM. ^#^ 324 RD cases also had CM. CIs by Wald.

**N. Investigation of the sickle trait/α^+^thalassaemia negative epistatic interaction and the *Sl2*/α^+^thalassaemia interaction by clinical outcome in the Kenyan case-control study.**

|  | **Sickle trait^*^** | | **α^+^thalassaemia^*^** | | **α^+^thalassaemia/**  **sickle trait**  **interaction^*^** | |  | ***Sl2^#^*** | | **α^+^thalassaemia*^#^*** | | **α^+^thalassaemia/ *Sl2* interaction*^#^*** | |
| --- | --- | --- | --- | --- | --- | --- | --- | --- | --- | --- | --- | --- | --- |
|  | **aOR**  **(95% CI)** | **p value** | **aOR**  **(95% CI)** | **p value** | **Interaction effect**  **(95% CI)** | **p value** |  | **aOR**  **(95% CI)** | **p value** | **aOR**  **(95% CI)** | **p value** | **Interaction effect**  **(95% CI)** | **p value** |
| **All severe malaria (n=1716)** | 0.11  (0.07-0.16) | <0.001 | 0.73  (0.61-0.88) | <0.001 | 3.16  (1.50-6.68) | 0.003 |  | 0.77  (0.63-0.94) | 0.010 | 0.73  (0.62-0.86) | <0.001 | 1.25  (0.97 – 1.60) | 0.086 |
| **CM**  **(n= 943)** | 0.12  (0.08-0.20) | <0.001 | 0.67  (0.53-0.84) | <0.001 | 1.79  (0.57-5.59) | 0.316 |  | 0.68  (0.53-0.87) | 0.002 | 0.73  (0.60-0.89) | 0.002 | 1.41  (1.03 – 1.93) | 0.031 |
| **SMA**  **(n=483)** | 0.06  (0.03-0.15) | <0.001 | 0.60  (0.44-0.83) | 0.002 | 3.82  (0.71-20.59) | 0.119 |  | 0.75  (0.55-1.02) | 0.064 | 0.67  (0.52-0.87) | 0.003 | 1.20  (0.80-1.80) | 0.369 |
| **Severe without CM (n=674)** | 0.10  (0.05-0.18) | <0.001 | 0.81  (063-1.04) | 0.102 | 5.54  (2.04-15.03) | 0.001 |  | 0.98  (0.75-1.28) | 0.879 | 0.73  (0.57-0.92) | 0.007 | 0.98  (0.70-1.39) | 0.924 |

Coefficients and p values are for the sickle trait/α^+^thalassaemia interaction and the *Sl2*/α^+^thalassaemia interaction by clinical outcome in two separate models (denoted by * or #). The sickle trait/α^+^thalassaemia interaction (examined in the * model) was only significant for cases without cerebral malaria, whereas the *Sl2*/α^+^thalassaemia interaction (examined in the # model) was only significant for cases with cerebral malaria. Both models include ethnicity and location as random effects with additional variables described in the * or # paragraphs below (NB, *McC^b^* and ABO are not included in these models).

* This model included the α^+^thalassaemia variable in recessive form (i.e. α^+^thalassaemia homozygotes vs both α^+^thalassaemia heterozygotes and those without α^+^thalassaemia alleles), sickle cell trait (i.e. sickle cell trait vs both sickle cell homozygotes and those without sickle cell alleles) and an interaction term between these two variables. This is the negative epistatic interaction as described in Williams TN et al, Nature Genetics 2005;37:1253-7.

# This model included the *Sl2* variable in the recessive form (i.e. *Sl2*/*Sl2* genotype vs both *Sl1*/*Sl1* and *Sl1*/*Sl2* genotypes) and the α^+^thalassaemia variable in the dominant form (i.e. one or more α^+^thalassaemia alleles vs no α^+^thalassaemia alleles). This is the novel interaction identified by the current study.

The logistic regression interaction effect is interpreted as a ratio of odds ratios. For example, there is a significant association between α^+^thalassaemia homozygosity and sickle cell trait for severe malaria. The association between α^+^thalassaemia homozygosity and severe malaria for those WITHOUT sickle cell trait is aOR=0.73. The association between α+thalassaemia homozygosity and severe malaria for those WITH sickle cell trait is aOR=0.73 * 3.16 = 2.31.

CM = cerebral malaria; SMA = severe malarial anaemia. Confidence intervals by Wald. The 95% CIs and p values are not bootstrapped. 260/483 cases of SMA also had CM.

**O. Reanalysis of the Kenyan case-control study excluding all children with one or more sickle cell alleles.**

| **Clinical Outcome** | ***Sl2***  **aOR (95% CI)** | ***p value*** | ***McCb***  **aOR (95% CI)** | ***p value*** |
| --- | --- | --- | --- | --- |
| **All severe malaria**  **(n= 1665)** | **0.75**  **(0.61 - 0.93)** | **0.009** | 1.10  (0.97 - 1.24) | 0.154 |
| **CM**  **(n=913)** | **0.65**  **(0.50 - 0.84)** | **0.001** | **1.18**  **(1.01 - 1.37)** | **0.035** |
| **Severe without CM**  **(n= 654)** | 0.98  (0.74 - 1.29) | 0.867 | 0.97  (0.81 - 1.16) | 0.728 |
| **Died**  **(n= 170)** | **0.41**  **(0.24 - 0.71)** | **0.001** | 1.33  (0.99 - 1.79) | 0.061 |
| **SMA**  **(n=469)** | 0.74  (0.54-1.02) | 0.069 | 0.95  (0.78- 1.17) | 0.658 |
| **RD**  **(n= 409)** | 0.78  (0.57 - 1.07) | 0.126 | 1.12  (0.92 - 1.35) | 0.247 |

Analysis of the Kilifi case-control dataset restricted to children without sickle cell trait or sickle cell disease (664 children excluded from analysis).

Adjusted Odds Ratios (aOR) and 95% Confidence Intervals (CI) are presented for the *Sl2* genotype in the recessive form (i.e. *Sl2/Sl2* vs all other *Sl* genotypes) and *McC^b^* genotype in the additive form (i.e. change in aOR with each additional *McC^b^* allele). aORs displayed are adjusted for ethnicity, location of residence, α^+^thalassaemia genotype and ABO blood group. An interaction term between *Sl* genotype and α^+^thalassaemia was included in the model. Confidence intervals by Wald. The 95% CIs and p values are not bootstrapped.

CM = cerebral malaria; SMA = severe malarial anaemia; RD, respiratory distress.

**P. Raw data for the combined sickle trait, α^+^thalassaemia and *Sl* genotype by clinical outcome in the Kenyan case-control study.**

| **Sickle Trait** | **α^+^thalassaemia** | ***Sl2*** | **Controls** | **All severe cases** | **All cases Proportion**  **(%)** | **CM**  **cases** | **CM Proportion**  **(%)** | **SMA**  **cases** | **SMA Proportion**  **(%)** | **Case**  **no CM** | **Case no CM Proportion**  **(%)** |
| --- | --- | --- | --- | --- | --- | --- | --- | --- | --- | --- | --- |
| 0 | 0 | 0 | 103 | 68 | *39.77* | 35 | *25.36* | 27 | *20.77* | 29 | *21.97* |
| 0 | 0 | 1 | 495 | 325 | *39.63* | 186 | *27.31* | 94 | *15.96* | 120 | *19.51* |
| 0 | 0 | 2 | 504 | 275 | *35.30* | 133 | *20.88* | 81 | *13.85* | 131 | *20.63* |
| 0 | 1 | 0 | 161 | 85 | *34.55* | 51 | *24.06* | 24 | *12.97* | 28 | *14.81* |
| 0 | 1 | 1 | 740 | 356 | *32.48* | 205 | *21.69* | 107 | *12.63* | 131 | *15.04* |
| 0 | 1 | 2 | 714 | 363 | *33.70* | 207 | *22.48* | 95 | *11.74* | 132 | *15.60* |
| 0 | 2 | 0 | 56 | 22 | *28.21* | 7 | *11.11* | 2 | *3.45* | 14 | *20.00* |
| 0 | 2 | 1 | 232 | 101 | *30.33* | 55 | *19.16* | 26 | *10.08* | 39 | *14.39* |
| 0 | 2 | 2 | 243 | 80 | *24.77* | 42 | *14.74* | 20 | *7.60* | 32 | *11.64* |
| 1 | 0 | 0 | 17 | 1 | *5.56* | 1 | *5.56* | 0 | *0.00* | 0 | *0.00* |
| 1 | 0 | 1 | 87 | 5 | *5.43* | 4 | *4.40* | 0 | *0.00* | 1 | *1.14* |
| 1 | 0 | 2 | 104 | 7 | *6.31* | 4 | *3.70* | 1 | *0.95* | 3 | *2.80* |
| 1 | 1 | 0 | 32 | 1 | *3.03* | 1 | *3.03* | 0 | *0.00* | 0 | *0.00* |
| 1 | 1 | 1 | 139 | 9 | *6.08* | 6 | *4.14* | 2 | *1.42* | 2 | *1.42* |
| 1 | 1 | 2 | 113 | 6 | *5.04* | 2 | *1.74* | 2 | *1.74* | 4 | *3.42* |
| 1 | 2 | 0 | 9 | 0 | *0.00* | 0 | *0.00* | 0 | *0.00* | 0 | *0.00* |
| 1 | 2 | 1 | 37 | 5 | *11.90* | 1 | *2.63* | 1 | *2.63* | 4 | *9.76* |
| 1 | 2 | 2 | 43 | 7 | *14.00* | 3 | *6.52* | 1 | *2.27* | 4 | *8.51* |

Raw data for each clinical outcome presented by sickle trait status (0 = no sickle trait, 1 = sickle trait), α^+^thalassaemia genotype (0, 1, 2 = number of α^+^thalassaemia alleles) and *Sl2* genotype (0, 1, 2 = number of *Sl2* alleles).

Proportion for each clinical outcome calculated using number of cases / (number of cases + number of controls).

**Q. Correlations between the sickle cell,** **α+thalassaemia, *Sl2* and *McC^b^* variants in the Kenyan case-control study.**

|  | Sickle | α+thalassaemia | *Sl2* | *McC^b^* |
| --- | --- | --- | --- | --- |
| Sickle |  | 0.011 | 0.006 | -0.025 |
| α+thalassaemia | 0.011 |  | -0.007 | 0.002 |
| *Sl2* | 0.006 | -0.007 |  | 0.301 |
| *McC^b^* | -0.025 | 0.002 | 0.301 |  |

Each variant was coded as 0/1/2 copies of the allele. Shaded area = not applicable

**R. Adjusted incidence Rate Ratios (IRRs) for *Sl* disease associations in the longitudinal cohort study by genetic models of inheritance**

|  | **Sl2 recessive** | **Sl2 additive** | **Sl2 heterozygous** | **Sl2 dominant** | **Sl2 genotypic (Sl1/Sl1 = ref)**  **Sl1/Sl2 Sl2/Sl2** | |
| --- | --- | --- | --- | --- | --- | --- |
| **Uncomplicated malaria**  aIRR (95% CI)  p value | **0.44 (0.26-0.74)***  **0.002** | **0.49 (0.34-0.72)***  **<0.001** | 0.95 (0.71-1.28)  0.735 | **0.35 (0.16-0.74)***  **0.006** | 0.43 (0.20-0.93)* | **0.23 (0.10-0.52)***  **<0.001** |
| **All non-malaria clinic visits**  aIRR (95% CI)  p value | 1.13 (0.96-1.32)  0.140 | 1.07 (0.95-1.20)  0.271 | 0.90 (0.77-1.04)  0.148 | 1.00 (0.78-1.27)  0.965 | 0.95 (0.73-1.22)  0.667 | 1.07 (0.82-1.40)  0.602 |
| **LRTI**^†^  aIRR (95% CI)  p value | 1.09 (0.81-1.47)  0.561 | 1.06 (0.85-1.32)  0.629 | 0.93 (0.69-1.24)  0.608 | 1.03 (0.64-1.65)  0.918 | 0.98 (0.60-1.61)  0.938 | 1.08 (0.65-1.77)  0.777 |
| **URTI**^¶^  aIRR (95% CI)  p value | 1.21 (0.98-1.50)  0.073 | 1.12 (0.96-1.32)  0.148 | 0.84 (0.68-1.03)  0.098 | 1.03 (0.74-1.45)  0.844 | 0.94 (0.66-1.33)  0.732 | 1.15 (0.81-1.64)  0.431 |
| **Gastroenteritis**  aIRR (95% CI)  p value | 0.94 (0.69-1.28)  0.694 | 0.87 (0.69-1.09)  0.214 | 0.89 (0.66-1.18)  0.406 | 0.66 (0.43-1.03)  0.066 | 0.66 (0.42-1.04)  0.072 | 0.67 (0.41-1.08)  0.100 |
| **Skin infection**  aIRR (95% CI)  p value | 1.07 (0.78-1.48)  0.668 | 1.11 (0.87-1.42)  0.410 | 1.03 (0.75-1.42)  0.836 | 1.33 (0.79-2.26)  0.285 | 1.32 (0.76-2.29)  0.324 | 1.35 (0.77-2.36)  0.291 |
| **Helminth infection**  aIRR (95% CI)  p value | 1.15 (0.71-1.86)  0.577 | 1.25 (0.86-1.81)  0.234 | 1.10 (0.69-1.76)  0.676 | 1.98 (0.83-4.71)  0.122 | 1.95 (0.80-4.73)  0.141 | 2.04 (0.82-5.07)  0.127 |
| **Malaria-negative fever**  aIRR (95% CI)  p value | 0.91 (0.72-1.15)  0.419 | 0.91 (0.77-1.08)  0.274 | 1.01 (0.81-1.27)  0.910 | 0.84 (0.60-1.18)  0.314 | 0.87 (0.61-1.24)  0.424 | 0.81 (0.56-1.17)  0.257 |

Data were collected from 22 *Sl1/Sl1*, 94 *Sl1/Sl2* and 92 *Sl2/Sl2* individuals during 49.4, 213.8 and 188.8 cyfu (child-years of follow-up) respectively. *Sl2* was tested for association with the disease outcomes of interest in the recessive, additive, heterozygous, dominant, and genotypic models. Adjusted incidence rate ratios and 95% confidence intervals (CIs) were generated using Poisson regression analysis, with adjustment for *McC*, α^+^thalassaemia and sickle cell genotypes, ABO blood group, season (divided into 3 monthly blocks), ethnicity, age as a continuous variable and within-person clustering of events. ^§^aIRRs: adjusted Incidence Rate Ratios**. ***Models that showed evidence of significant interaction between *Sl* and α^+^thalassaemia. ^†^LRTI: Lower Respiratory Tract Infection. ^¶^URTI: Upper Respiratory Tract Infection. Statistically significant associations (P <0.05) are shown in bold.

**S. Adjusted incidence rate ratios for *McC* disease associations in the longitudinal cohort study by genetic models of inheritance**

|  | **McC^b^ recessive** | **McC^b^ additive** | **McC^b^ heterozygous** | **McC^b^ dominant** | **McC^b^ genotypic (McC^a^ / McC^a^ = ref)**  **McC^a^ / McC^b^ McC^b^ / McC^b^** | |
| --- | --- | --- | --- | --- | --- | --- |
| **Uncomplicated malaria**  aIRR (95% CI)  p value | 1.01 (0.44-2.34)  0.977 | 1.18 (0.90-1.56)  0.239 | 1.24 (0.90-1.70)  0.185 | 1.24 (0.90-1.69)  0.184 | 1.25 (0.91-1.72)  0.177 | 1.12 (0.48-2.60)  0.798 |
| **All non-malaria clinic visits**  aIRR (95% CI)  p value | 0.75 (0.50-1.13)  0.163 | **0.76 (0.61-0.96)***  **0.020** | 0.95 (0.80-1.12)  0.548 | **0.76 (0.57-0.99)***  **0.047** | 0.93 (0.79-1.10)  0.405 | 0.73 (0.48-1.10)  0.130 |
| **LRTI**^†^  aIRR (95% CI)  p value | **0.39 (0.16-0.96)**  **0.040** | 0.87 (0.65-1.15)  0.314 | 1.07 (0.77-1.50)  0.678 | 0.94 (0.68-1.30)  0.713 | 1.02 (0.73-1.42)  0.913 | **0.39 (0.16-0.97)**  **0.043** |
| **URTI**^¶^  aIRR (95% CI)  p value | 1.08 (0.63-1.87)  0.772 | 0.87 (0.72-1.05)  0.148 | **0.79 (0.63-0.99)**  **0.047** | 0.81 (0.65-1.01)  0.068 | **0.79 (0.63-0.99)**  **0.049** | 0.99 (0.57-1.72)  0.975 |
| **Gastroenteritis**  aIRR (95% CI)  p value | 0.78 (0.38-1.62)  0.501 | 0.65 (0.42-1.01)*  0.053 | 0.58 (0.31-1.07)*  0.083 | **0.55 (0.31-0.97)***  **0.038** | 1.02 (0.73-1.42)  0.906 | 0.78 (0.38-1.64)  0.516 |
| **Skin infection**  aIRR (95% CI)  p value | 0.42 (0.16-1.13)  0.086 | 1.01 (0.75-1.35)  0.974 | 1.25 (0.89-1.76)  0.202 | 1.12 (0.80-1.56)  0.520 | 1.21 (0.86-1.70)  0.281 | 0.45 (0.17-1.21)  0.114 |
| **Helminth infection**  aIRR (95% CI)  p value | 0.34 (0.04-2.70)  0.308 | 0.68 (0.43-1.07)  0.094 | 0.73 (0.44-1.22)  0.232 | 0.68 (0.41-1.12) | 0.71 (0.43-1.18)  0.189 | 0.31 (0.04-2.45)  0.266 |
| **Malaria-negative fever**  aIRR (95% CI)  p value | 0.95 (0.52-1.73)  0.870 | 1.01 (0.82-1.25)  0.926 | 1.03 (0.80-1.33)  0.802 | 1.02 (0.80-1.32)  0.858 | 1.03 (0.80-1.33)  0.822 | 0.96 (0.52-1.77)  0.904 |

Data were collected from 137 *McC^a^/McC^a^*, 63 *McC^a^*/*McC^b^* and 8 *McC^b^/McC^b^* individuals during 294.5, 143.2 and 14.3 cyfu (child-years of follow-up) respectively. *McC^b^* was tested for association with the disease outcomes of interest in the recessive, additive, heterozygous, dominant, and genotypic models. Adjusted incidence rate ratios and 95% confidence intervals (CIs) were generated using Poisson regression analysis, with adjustment for *Sl*, α^+^thalassaemia and sickle cell genotypes, ABO blood group, season (divided into 3 monthly blocks), ethnicity, age as a continuous variable and within-person clustering of events. ^§^aIRRs: adjusted Incidence Rate Ratios**. ***Models that showed evidence of significant interaction between *McC* and α^+^thalassaemia. ^†^LRTI: Lower Respiratory Tract Infection. ^¶^URTI: Upper Respiratory Tract Infection. Statistically significant associations (P <0.05) are shown in bold.
