# Supplementary material for: Two complement receptor one alleles have opposing associations with cerebral malaria and interact with α+thalassaemia
Source: eLife. 2018 Apr 25;7:e31579. doi: 10.7554/eLife.31579 (PMC5953541; doi:10.7554/eLife.31579)
Supplement: Reporting standard 1 [file elife-31579-fig2.doc]

STROBE Statement—checklist of items that should be included in reports of observational studies

STREGA Statement – an extension to the STROBE checklist for genetic association studies

**Paper Title: Two Complement Receptor One alleles have opposing associations with cerebral malaria and interact with α^+^thalassaemia**

|  | Item No | STROBE | STREGA |
| --- | --- | --- | --- |
| **Title and abstract** | 1 | (*a*) Indicate the study’s design with a commonly used term in the title or the abstract  This has been indicated in the **abstract** |  |
|  |  | (*b*) Provide in the abstract an informative and balanced summary of what was done and what was found  The **abstract** gives a summary interpretation of the methods and findings. |  |
| Introduction | | |  |
| Background/rationale | 2 | Explain the scientific background and rationale for the investigation being reported  **Introduction paragraph 2** |  |
| Objectives | 3 | State specific objectives, including any prespecified hypotheses  **Introduction paragraph 2** | State if the study is the first report of a genetic association, a replication effort, or both.  **Both – introduction paragraph 2** |
| Methods | | |  |
| Study design | 4 | Present key elements of study design early in the paper  **Materials and methods paragraphs 1-7** |  |
| Setting | 5 | Describe the setting, locations, and relevant dates, including periods of recruitment, exposure, follow-up, and data collection  **Materials and methods paragraphs 1, 2, 3, 6 and 7** |  |
| Participants | 6 | (*a*) *Cohort study*—Give the eligibility criteria, and the sources and methods of selection of participants. Describe methods of follow-up  **Material and methods paragraph 6**  *Case-control study*—Give the eligibility criteria, and the sources and methods of case ascertainment and control selection. Give the rationale for the choice of cases and controls  **Material and methods paragraphs 3 and 7, Figure 3 and appendix 1 Figure 1**  *Cross-sectional study*—Give the eligibility criteria, and the sources and methods of selection of participants  **N/A** | Give information on the criteria and methods for selection of subsets of participants from a larger study, when relevant.  **Materials and Methods paragraphs 3, 6 and 7, and Figure 3 and appendix 1 Figure 1** |
|  |  | (*b*) *Cohort study*—For matched studies, give matching criteria and number of exposed and unexposed  **N/A**  *Case-control study*—For matched studies, give matching criteria and the number of controls per case  **N/A** |  |
| Variables | 7 | a) Clearly define all outcomes, exposures, predictors, potential confounders, and effect modifiers. Give diagnostic criteria, if applicable  **Material and methods paragraphs 3, 5, 6, 7 and 9** | b) Clearly define genetic exposures (genetic variants) using a widely-used nomenclature system. Identify variables likely to be associated with population stratification (confounding by ethnic origin).  **Material and methods paragraphs 3, 4, 5, 6, 7 and 9 (Laboratory procedures)** |
| Data sources/ measurement | 8* | a) For each variable of interest, give sources of data and details of methods of assessment (measurement). Describe comparability of assessment methods if there is more than one group | b) Describe laboratory methods, including source and storage of DNA, genotyping methods and platforms (including the allele calling algorithm used, and its version), error rates and call rates. State the laboratory/centre where genotyping was done. Describe comparability of laboratory methods if there is more than one group. Specify whether genotypes were assigned using all of the data from the study simultaneously or in smaller batches.  **Materials and methods paragraphs 4, 8 and 9** |
| Bias | 9 | a) Describe any efforts to address potential sources of bias  **N/A** | b) For quantitative outcome variables, specify if any investigation of potential bias resulting from pharmacotherapy was undertaken. If relevant, describe the nature and magnitude of the potential bias, and explain what approach was used to deal with this.  **N/A** |
| Study size | 10 | Explain how the study size was arrived at  **Study size was pragmatic, based on all cases of severe malaria presenting during the study period.** |  |
| Quantitative variables | 11 | Explain how quantitative variables were handled in the analyses. If applicable, describe which groupings were chosen and why  **Material and methods paragraph 10 (Statistical analysis), appendix 2 (Detailed statistical methods)** | If applicable, describe how effects of treatment were dealt with.  **N/A** |
| Statistical methods | 12 | (*a*) Describe all statistical methods, including those used to control for confounding  **Materials and methods paragraph 10 and appendix 2** | State software version used and options (or settings) chosen.  **Materials and Methods paragraph 10 and appendix 2**  f) State whether Hardy-Weinberg equilibrium was considered and, if so, how.  **Yes – Supplementary file 1C** |
|  |  | (*b*) Describe any methods used to examine subgroups and interactions  **Materials and methods paragraph 10 and appendix 2** | g) Describe any methods used for inferring genotypes or haplotypes.  **N/A** |
|  |  | (*c*) Explain how missing data were addressed | h) Describe any methods used to assess or address population stratification.  **Materials and methods paragraph 10 and appendix 2** |
|  |  | (*d*) *Cohort study*—If applicable, explain how loss to follow-up was addressed  **N/A**  *Case-control study*—If applicable, explain how matching of cases and controls was addressed  **N/A**  *Cross-sectional study*—If applicable, describe analytical methods taking account of sampling strategy  **N/A** | i) Describe any methods used to address multiple comparisons or to control risk of false positive findings.  **N/A** |
|  |  | (*e*) Describe any sensitivity analyses  **N/A** | j) Describe any methods used to address and correct for relatedness among  subjects.  **N/A** |
|  |  |  |  |

| Results | | |  |
| --- | --- | --- | --- |
| Participants | 13* | (a) Report numbers of individuals at each stage of study—eg numbers potentially eligible, examined for eligibility, confirmed eligible, included in the study, completing follow-up, and analysed  **Figure 3 and Appendix 1 Figure 1** | Report numbers of individuals in whom genotyping was attempted and numbers of individuals in whom genotyping was successful.  **Figure 3 and Appendix 1 Figure 1** |
|  |  | (b) Give reasons for non-participation at each stage  **Figure 3 and Appendix 1 Figure 1** |  |
|  |  | (c) Consider use of a flow diagram  **Figure 3 and Appendix 1 Figure 1** |  |
| Descriptive data | 14* | (a) Give characteristics of study participants (eg demographic, clinical, social) and information on exposures and potential confounders  **Supplementary Files 1A, 1B, 1G and Appendix 1 Table 2** | Consider giving information by genotype.  **Supplementary Files 1A, 1B, 1G and Appendix 1 Table 2** |
|  |  | (b) Indicate number of participants with missing data for each variable of interest  **Figure 3 and Appendix 1 Figure 1** |  |
|  |  | (c) *Cohort study*—Summarise follow-up time (eg, average and total amount)  **Table 2** |  |
| Outcome data | 15* | *Cohort study*—Report numbers of outcome events or summary measures over time  **Table 3 and Supplementary Files 1H and 1I** | Report outcomes (phenotypes) for each genotype category.  **Supplementary Files 1H and 1I** |
|  |  | *Case-control study—*Report numbers in each exposure category, or summary measures of exposure  **Supplementary Files 1A and 1B** | Report numbers in each genotype category.  **Supplementary File 1B** |
|  |  | *Cross-sectional study—*Report numbers of outcome events or summary measures  **N/A** | Report (outcomes) for each genotype  **N/A** |
| Main results | 16 | (*a*) Give unadjusted estimates and, if applicable, confounder-adjusted estimates and their precision (eg, 95% confidence interval). Make clear which confounders were adjusted for and why they were included  Unadjusted estimates are presented in **Supplementary Files 1D and 1J**  Adjusted estimates and their precisions and confounders are presented in **Tables 1 and 2, Results section paragraphs 1-5 and Supplementary Files 1E, 1F, 1K-1O, 1R and 1S** |  |
|  |  | (*b*) Report category boundaries when continuous variables were categorized  **N/A** |  |
|  |  | (*c*) If relevant, consider translating estimates of relative risk into absolute risk for a meaningful time period  **N/A** | d) Report results of any adjustments for multiple comparisons  **N/A** |
| Other analyses | 17 | a) Report other analyses done—eg analyses of subgroups and interactions, and sensitivity analyses  **Appendix 2** | b) If numerous genetic exposures (genetic variants) were examined, summarize results from all analyses undertaken.  **N/A**  c) If detailed results are available elsewhere, state how they can be accessed.  **N/A** |
| Discussion | | |  |
| Key results | 18 | Summarise key results with reference to study objectives  **Discussion paragraph 1** |  |
| Limitations | 19 | Discuss limitations of the study, taking into account sources of potential bias or imprecision. Discuss both direction and magnitude of any potential bias  **Discussion paragraph 6** |  |
| Interpretation | 20 | Give a cautious overall interpretation of results considering objectives, limitations, multiplicity of analyses, results from similar studies, and other relevant evidence  **Discussion paragraphs 2-5 and 7** |  |
| Generalisability | 21 | Discuss the generalisability (external validity) of the study results  **Discussion paragraph 7** |  |
| Other information | | |  |
| Funding | 22 | Give the source of funding and the role of the funders for the present study and, if applicable, for the original study on which the present article is based  **Funding and Role of funding source sections** |  |

*Give information separately for cases and controls in case-control studies and, if applicable, for exposed and unexposed groups in cohort and cross-sectional studies.

**Note:** An Explanation and Elaboration article discusses each checklist item and gives methodological background and published examples of transparent reporting. The STROBE checklist is best used in conjunction with this article (freely available on the Web sites of PLoS Medicine at http://www.plosmedicine.org/, Annals of Internal Medicine at http://www.annals.org/, and Epidemiology at http://www.epidem.com/). Information on the STROBE Initiative is available at www.strobe-statement.org.
