# Supplementary material for: Two complement receptor one alleles have opposing associations with cerebral malaria and interact with α+thalassaemia
Source: eLife. 2018 Apr 25;7:e31579. doi: 10.7554/eLife.31579 (PMC5953541; doi:10.7554/eLife.31579)
Supplement: Transparent reporting form [file elife-31579-transrepform.docx]

***eLife’s* transparent reporting form**

We encourage authors to provide detailed information *within their submission* to facilitate the interpretation and replication of experiments. Authors can upload supporting documentation to indicate the use of appropriate reporting guidelines for health-related research (see [EQUATOR Network](http://www.equator-network.org/%20)), life science research (see the [BioSharing Information Resource](https://biosharing.org/)), or the [ARRIVE guidelines](http://www.plosbiology.org/article/info:doi/10.1371/journal.pbio.1000412) for reporting work involving animal research. Where applicable, authors should refer to any relevant reporting standards documents in this form.

If you have any questions, please consult our Journal Policies and/or contact us: [editorial@elifesciences.org](mailto:editorial@elifesciences.org).

**Sample-size estimation**

- You should state whether an appropriate sample size was computed when the study was being designed
- You should state the statistical method of sample size computation and any required assumptions
- If no explicit power analysis was used, you should describe how you decided what sample (replicate) size (number) to use

Please outline where this information can be found within the submission (e.g., sections or figure legends), or explain why this information doesn’t apply to your submission:

Sample size of the case-control study was pragmatic, based on all cases of severe malaria presenting to during the study period. For recruitment details of case-control and cohort studies, please see Materials and Methods, line numbers 326-343 and 385-406.

**Replicates**

- You should report how often each experiment was performed
- You should include a definition of biological versus technical replication
- The data obtained should be provided and sufficient information should be provided to indicate the number of independent biological and/or technical replicates
- If you encountered any outliers, you should describe how these were handled
- Criteria for exclusion/inclusion of data should be clearly stated
- High-throughput sequence data should be uploaded before submission, with a private link for reviewers provided (these are available from both GEO and ArrayExpress)

Please outline where this information can be found within the submission (e.g., sections or figure legends), or explain why this information doesn’t apply to your submission:

For the ex-vivo rosetting assay (see Materials and Methods, line numbers 421-429), 167 *P. falciparum* isolates were studied, each of which can be considered a biological replicate representing the different Knops genotypes. The rosette frequency of each isolate was determined by making two wet preparations (technical replicates) from each isolate, and counting 100 infected red cells on each wet preparation. The overall rosette frequency for the isolate is the mean of the two counts.   All parasite isolates that matured to the pigmented trophozoite stage in the first asexual cycle in vitro were included in the original study (reference (21) (Doumbo OK et al. Am J Trop Med Hyg. 2009;81(6):987-93), and all isolates for whom Knops genotyping was successfully performed are included here.

**Statistical reporting**

- Statistical analysis methods should be described and justified
- Raw data should be presented in figures whenever informative to do so (typically when N per group is less than 10)
- For each experiment, you should identify the statistical tests used, exact values of N, definitions of center, methods of multiple test correction, and dispersion and precision measures (e.g., mean, median, SD, SEM, confidence intervals; and, for the major substantive results, a measure of effect size (e.g., Pearson's r, Cohen's d)
- Report exact p-values wherever possible alongside the summary statistics and 95% confidence intervals. These should be reported for all key questions and not only when the p-value is less than 0.05.

Please outline where this information can be found within the submission (e.g., sections or figure legends), or explain why this information doesn’t apply to your submission:

Please see “Statistical Analysis” in Materials and Methods section of the main manuscript (line numbers 444-486) and also Appendix 2 (“Detailed statistical methods”).

(For large datasets, or papers with a very large number of statistical tests, you may upload a single table file with tests, Ns, etc., with reference to sections in the manuscript.)

**Group allocation**

- Indicate how samples were allocated into experimental groups (in the case of clinical studies, please specify allocation to treatment method); if randomization was used, please also state if restricted randomization was applied
- Indicate if masking was used during group allocation, data collection and/or data analysis

Please outline where this information can be found within the submission (e.g., sections or figure legends), or explain why this information doesn’t apply to your submission:

Please see “The Kenyan case-control study” (line numbers 326-343) and “The Kenyan longitudinal cohort study” in Material and Methods section (line numbers 385-406).

**Additional data files (“source data”)**

- We encourage you to upload relevant additional data files, such as numerical data that are represented as a graph in a figure, or as a summary table
- Where provided, these should be in the most useful format, and they can be uploaded as “Source data” files linked to a main figure or table
- Include model definition files including the full list of parameters used
- Include code used for data analysis (e.g., R, MatLab)
- Avoid stating that data files are “available upon request”

Please indicate the figures or tables for which source data files have been provided:

This study uses data from the MalariaGen Consortial Project 1.

Publicly available at MalariaGen Genomic Epidemiology Network (www.malariagen.net).
